# Heat Flow in Solvent–Free, Dense Amorphous and Semi–Crystalline Cellulose Derivatives

**DOI:** 10.1021/acs.macromol.5c02281

**Published:** 2026-01-04

**Authors:** Debashish Mukherji, Tiago Espinosa de Oliveira, Nusrat Chowdhury, David G. Cahill, Marcus Müller

**Affiliations:** † Institut für Theoretische Physik, 9375 George−August−Universität Göttingen, 37077 Göttingen, Germany; ‡ Departamento de Farmacociências, 117303 Universidade Federal de Ciências da Saúde de Porto Alegre, Porto Alegre 90050−170, Brazil; § Department of Machanical Science and Engineering, 14589 University of Illinois Urbana−Champaign, Urbana, Illinois 61801, United States; ∥ Materials Research Laboratory, University of Illinois Urbana−Champaign, Urbana, Illinois 61801, United States; ⊥ Department of Materials Science and Engineering, University of Illinois Urbana−Champaign, Urbana, Illinois 61801, United States

## Abstract

Polymers are essential in our everyday life due to their versatility and tunable properties, but common synthetic polymers pose significant environmental challenges. This has led to growing interest in natural, biodegradable alternatives such as cellulose. For cellulose to serve as a viable alternative, it must match or ideally exceed materials properties of synthetic polymers. Thermal conductivity, κ, is one such critical property that often determines the suitability of polymers for a wide range of applications. In this study, we employ large–scale molecular dynamics simulations to investigate heat transport in dense, solvent–free cellulose and cellulose acetate systems. Our focus is on the amorphous phases of both materials, as well as the semi–crystalline phase of pure cellulose. By analyzing the vibrational density of states, *g*(ν), we report quantum–corrected estimates of the heat capacity, *c*, and consequently κ, enabling reasonable comparison with experimental data. Our results show that, over the temperature, *T*, range of 280–400 K, κ of amorphous cellulose varies between approximately 0.14 and 0.26 Wm^–1^ K^–1^, while slightly lower values, around 0.12 to 0.22 Wm^–1^ K^–1^, are observed for amorphous cellulose acetate. Within a similar temperature range, our experimental data for amorphous cellulose acetate give κ ≃ 0.15–0.21 Wm^–1^ K^–1^. In semi–crystalline cellulose samples, depending on *T*, κ can increase by approximately 20–35% when the degree of crystallinity reaches *d* ≃ 20%. These values are comparable to those of standard synthetic polymers, highlighting cellulose as a promising alternative. This study demonstrates that cellulose offers a natural, sustainable alternative to common synthetic polymers, while also providing insight into the thermal behavior of cellulose–based materials.

## Graphical abstract

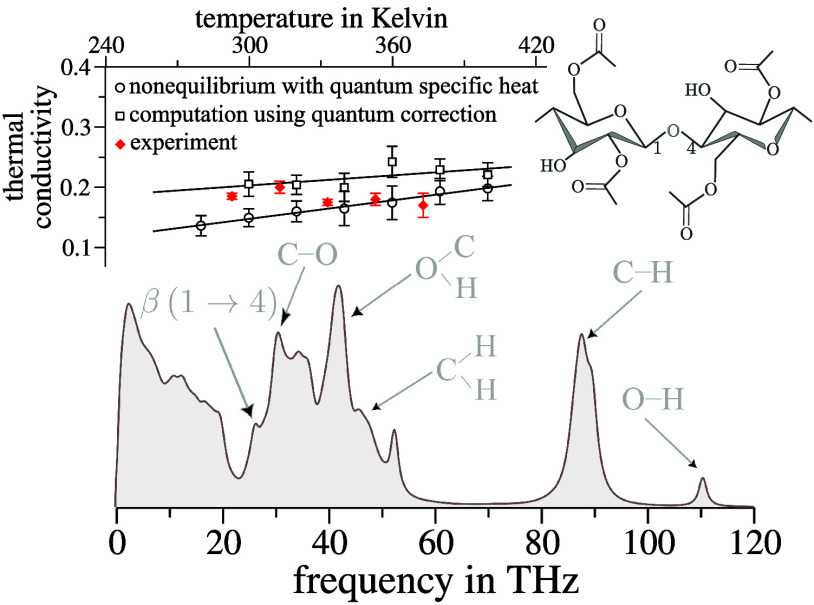

## 1 Introduction

A new era began with Hermann Staudinger’s pioneering work,^1^ which laid the foundation for the field of modern polymer science. Since then, polymer science has made significant advances, with polymers now playing a crucial role in a wide range of applications.^2–7^ Typically, a polymer architecture consists of stiff carboncarbon (C–C) backbone bonds, angular, and dihedral interactions, which maintains its structure, while the microscopic, softer non–bonded interactions determine its material properties. Typical examples of such non–bonded interactions are hydrogen bonding (H–bond) and van der Waals (vdW), the strength of which are 4–8*k*_B_ *T* and *k*_B_ *T*,^6,8^ respectively. Here, *k*_B_ is the Boltzmann constant and the energy scales are estimated with respect to ambient temperature (*T* = 300 K). On the other hand, C–C bonds are exceptionally strong, with their strength exceeding 80*k*_B_ *T*. The major drawback of such bonds is that they do not degrade naturally, leading to increasing environmental concerns. This has motivated to a shift in focus on natural alternatives. Among the various natural alternatives, polysaccharides emerge as a particularly promising candidate due to their abundance, biodegradability, sustainability, and scalability in production.^2,3,7^

Polysaccharides are long–chain macromolecules composed of d–glucose units connected by ether (C–O–C) linkages, typically through a β(1 → 4) glycosidic bond (see Figure 1). These natural polymers are inherently biodegradable. A well–known example of polysaccharide is cellulose, in which the side group is a hydrogen atom (see Figure 1a). Cellulose is a fundamental macromolecular component of the wall of plant cells, forming microfibrils– also known as cellulose fibrils (CF). These microfibrils are composed of tightly packed cellulose molecules, held together by H–bond between hydrophilic side groups and vdW interactions among stacked d–glucose units. This arrangement gives cellulose fibrils a hydrophilic surface and a highly cohesive, strongly interacting core.

**Figure 1:**
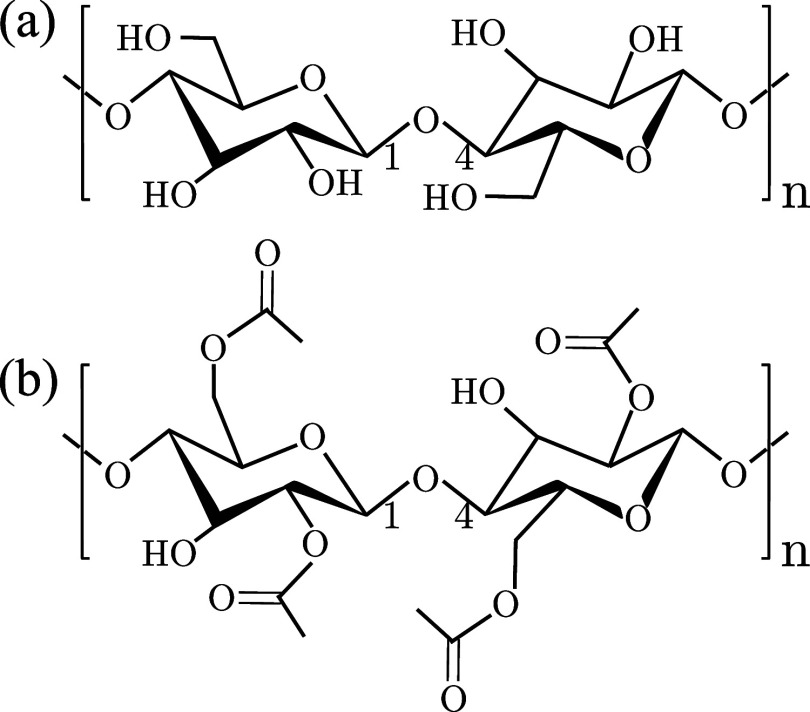
Schematics illustrating typical polysaccharide derivatives are shown. Parts (a, b) depict the structures of cellulose and cellulose acetate, respectively. In cellulose, the side group attached to the d–glucose ring is a hydrogen (H) atom. In cellulose acetate, this side group is replaced by an acetyl group, −C­(=O)–CH_3_.

Cellulose naturally tends to form crystalline structures, which can be undesirable for applications requiring amorphous, flexible plastics. To address this, the side groups on the glucose units can be chemically modified to disrupt crystallinity and adjust the solubility, structure, and functional properties of the polysaccharide chain. A widely used example of such a modified cellulose is cellulose acetate, in which the hydroxyl groups are substituted with acetyl groups −C­(=O)–CH_3_ (see Figure 1b).

To develop cellulose–based materials with desirable physical properties, these chains are routinely processed in a variety of solvents.^9–11^ Common materials derived from cellulose include nanocrystals,^2,3,7^ fibers,^12^ cellulose papers,^13^ aerogels,^14–17^ and cellophane.^18^ These materials are often subjected to diverse environmental conditions – such as varying solution processing, mechanical stability, temperature, and pressure – which influence their morphology and material properties.

One of the most critical properties determining a material’s utility is its thermal conductivity, quantified by the thermal transport coefficient κ.^19–22^ For instance, materials with low κ values are well–suited for thermal insulation, as seen in aerogels.^14–17^ In contrast, high power density applications require materials with high κ to facilitate efficient heat dissipation.^23,24^ Consequently, recent research has increasingly focused on understanding the κ–behavior of cellulose. However, most studies to date have been experimental, revealing a wide range of κ values depending on the cellulose structure and processing methods. In particular, cellulose aerogels–depending on solution processing and void architecture–have reported κ values between 0.018 and 0.070 Wm^–1^ K^–1^;^14–17^ cellulose papers exhibit κ = 0.076–0.121 Wm^–1^ K^–1^ over the temperature range of *T* = 298–373 K; and cellulose fibers have reported a maximum in κ ≃ 14.50 Wm^–1^ K^–1^.^12^ Randomly oriented cellulose nanocrystal (CNC) networks reported κ ≃ 0.60 Wm^–1^ K^–1^,^25^ while CNCs in a liquid–crystalline arrangement exhibit κ values ranging from 0.22–0.53 Wm^–1^ K^–1^.^26^

While experimental studies have provided intriguing insights, simulation data on cellulose^26,27^ and its derivatives remain relatively limited. Notably, these simulation studies have addressed κ calculations in nematic assemblies of nanocrystalline cellulose,^26^ as well as in assemblies of short cellulose oligomers with lengths of roughly two persistence lengths, $l_{p}$ .^27^ This lack in simulation studies arises from several challenges: (1) Cellulose is a comparably stiff molecule, with a typical $l_{p}≃3.4\;\mathrm{nm}$ –equivalent to about six d–glucose units (see Figure 1) – whereas commodity polymers such as poly­(methyl methacrylate) (PMMA) have a significantly shorter $l_{p}≃0.72\;\mathrm{nm}$ .^28,29^ (2) The increased stiffness of cellulose necessitates the simulation of significantly longer chain lengths $N_{l}$ , which in turn leads to much longer molecular–relaxation time scales. (3) Additionally, simulating these long $N_{l}$ requires correspondingly larger system sizes to avoid finite–size effects, further increasing computational demands. To bridge the gap between theoretical predictions and experimental observations, we conduct large–scale, unbiased molecular dynamics simulations. These simulations involve systems containing 0.63–0.93 million particles and span a cumulative simulation time exceeding 11 μs. Our focus is on investigating thermal transport in high–density amorphous cellulose and cellulose acetate, as well as moderately semi–crystalline cellulose samples. Using quantum correction previously proposed by one of us,^30,31^ we report quantum–corrected estimates of κ. These values serve as bounds that can be compared with experimental results. For this purpose, we have also performed experimental measurements on amorphous cellulose acetate samples to enable a direct quantitative comparison between our simulation results and the experimental data. These results may help guide the design of next–generation cellulose-based materials with tunable thermal transport properties.

The remainder of our paper is organized as follows: Section 2 outlines the materials and simulation methodology and experimental details are highlighted in Section 3. In Section 4, we revisit key concepts related to heat flow in polymers and the fundamentals of polymer thermal conductivity. Section 5 presents our results for both amorphous and semi–crystalline cellulose materials, along with a discussion of potential strategies for tuning κ. Finally, the conclusions are summarized in Section 6.

## 2 Materials, Model, and Method

In this study, we investigate an amorphous cellulose and cellulose acetate samples (see Figure 1) and a set of semi–crystalline cellulose samples with varying degree, *d*, of crystallinity. In all cases, each polymer chain is comprised of $N_{l}=50$ d–glucose units (equivalent to *n* = 25 monomer repeat units in Figure 1), which corresponds to approximately 8–10 persistence length segments per chain.

A key structural feature of a d–glucose ring in cellulose is its atomic structure. Notably, d–glucose can adopt two primary conformations: the chair and the boat. Here, the free–energy difference between the boat and chair conformations is around 10*k*_B_ *T*, while the free–energy barrier between these two states is approximately 8*k*_B_ *T*. The chair conformation (highlighted in Figure 1) is thermodynamically more stable in cellulose, influencing both its solvation behavior and self–assembly, and thus its material properties. To accurately capture this structure, we employ the GLYCAM06 force field^32,33^ for both cellulose samples, which is a highly reliable atomistic model, known to reproduce the chair conformation of d–glucose units. Simulations are carried out using the GROMACS simulation package.^34^

Initial configurations are generated by randomly placing *N*_c_ = 601 chains into a cubic simulation box. The box has a lateral dimension of approximately *L* ≃ 20 nm for cellulose and *L* ≃ 24 nm for cellulose acetate, resulting in total system sizes of roughly *N* ≃ 0.63 million atoms for cellulose and *N* ≃ 0.93 million atoms for cellulose acetate.

The configurations are equilibrated in the isothermal–isobaric (*N*_p_ *T*) ensemble their melt states at a temperature *T* = 800 K. Here, *T* is controlled using the canonical sampling by velocity–rescaling thermostat (v–rescale)^35^ with a time constant τ_T_ = 1 ps. We maintain a constant pressure of 1 atm using the stochastic cell rescaling barostat (c–rescale)^36^ with a pressure coupling time of τ_p_ = 0.5 ps. Note that pressure coupling using c–rescale together with v–rescale samples configurations according to the *N*_p_ *T* ensemble. After this equilibration stage, the property calculations are performed using the langevin thermostat. Details will be discussed whenever appropriate.

Long–range electrostatic interactions are handled using the particle–mesh Ewald (PME) method,^37^ and non–bonded interactions are truncated at a cutoff distance of *r*_c_ = 1.0 nm. The equations of motion are integrated using the leap–frog algorithm.^38^ Unless states otherwise, the integration time step Δ*t* is chosen as 1 fs during the equilibration stage and 0.1 fs for the property calculations.

### 2.1 Sample Preparation

Both cellulose systems were first equilibrated for 2 μs, with stiff bond vibrations constrained using the LINCS algorithm,^39^ allowing for a larger integration time step of Δ*t* = 1 fs. During this stage, cellulose acetate reached equilibrium, as its relaxation time – defined as the time required for a chain to diffuse a length comparable to its own end–to–end distance *R*_ee_ ≃ 8.8 nm – is approximately 70 ns. Moreover, in the pure cellulose sample, we observe a progressive straightening of individual chains, indicating the onset of crystallization. To capture this structural evolution and ensure proper equilibration, the system was simulated for an additional 1 μs, resulting in a total equilibration time of 3 μs.

To further investigate the crystallization process in the pure cellulose sample, we computed the collective scattering function using

$$S\left(k\right)=\frac{1}{N_{o}}\left\langle {\left|\overset{N_{o}}{\underset{i=1}{\sum }}e^{\mathrm{ik}\cdot R_{i}}\right|}^{2}\right\rangle$$ 1

where *N*_0_ denotes the number of scatters at positions, **R**_*i*_ with *i* = 1,···,*N*. For the calculation of *S*(*k*) only the positions of the two β(1 → 4) oxygen linker in each monomeric repeat unit are considered, giving *N*_0_ = 30,050. This includes terminal oxygen atoms at the chain ends.

Figure 2(a) presents *S*(*k*) at various times. The growing intensity of the Bragg peaks with time provides evidence of a progressive increase in crystallinity. Additionally, we note that the two prominent correlation peaks in Figure 2(a) correspond to (i) the intrachain peak between the nearest neighbor (bonded) β(1 → 4) oxygen linker at *k* ≃ 10.5 nm^–1^ (corresponding to 2π/*k* ≃ 0.60 nm) and (ii) the interchain (nonbonded) nearest–neighbor peak at *k* ≃ 14.5 nm^–1^ (corresponding to 2π/*k* ≃ 0.43 nm).

**Figure 2:**
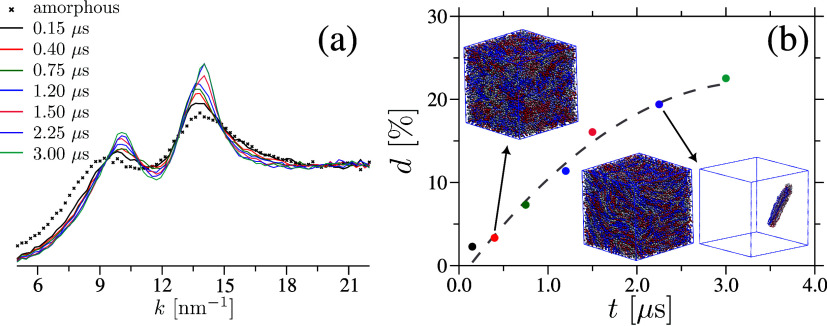
Main panel (a) displays the scattering function *S*(*k*) of pure cellulose as a function of wavenumber *k*, presented at various simulation times. Panel (b) highlights the degree of crystallinity *d*, illustrating its evolution over time. Simulation snapshots in panel (b) highlight the chain orientation and crystallization observed within the sample for both *d* values. For *d* = 20%, we have also highlighted the largest crystalline domain within a sample. Note that only the oxygen atoms in the β(1 → 4) glycosidic bonds is taken for the calculation and also shown in the snapshots.

While the protocol described above is used to generate semi–crystalline cellulose samples, a fully amorphous sample is prepared using a different approach. Specifically, a configuration obtained after 2 μs at *T* = 800 K is used as the starting point for an additional simulation at *T* = 1000 K for another *t* ≃1.5 μs. In this completely amorphous sample, $l_{p}$ is calculated to be approximately 3.4 nm (corresponding to about 6–7 d–glucose units). The corresponding *S*(*k*) – represented by the × data set in Figure 2(a) – exhibits smaller Bragg–peak intensities, confirming the loss of crystalline order in this amorphous cellulose sample. It is worth noting, however, that due to the inherently large $l_{p}$ of cellulose chains, some degree of segmental stacking persists even in the amorphous state, as evidenced by the residual intensities of the broad Bragg peaks in Figure 2(a). Such an segmental stacking is hindered due to the presence of a relatively longer and flexible side group in cellulose acetate.

The degree of crystallinity, *d*, is typically estimated from the structure factor *S*(*k*). However, accurately determining *d* requires careful subtraction of the amorphous background, which can be challenging in samples with complex molecular architectures. Therefore, to obtain reliable estimates of *d* within a sample, we employed a protocol similar to – but slightly modified from – that described in ref 40. The procedure is as follows:

(a) Two successive segments of length $l_{p}$ are selected along a cellulose backbone. The second Legendre polynomial *P*_2_(*i*, *i* + 1) = {3 cos^2^(θ_*i*,*i*+1_) – 1}/2 is calculated between them, where θ_*i*,*i*+1_ is the angle between segments 1 and 2.
(b) If *P*_2_(1, 2) ≥ 0.95 (or θ_1, 2_ ≤ 10°), then the alignment is considered crystal–like aligned, and *P*_2_(2, 3) is calculated for the next pair of successive segments. Note that the criterion *P*_2_(1, 2) ≥ 0.95 corresponds to the position of the full–width–at–half–maximum (fwhm) in the distribution of *P*_2_, which is sharply peakes around 1.^40^
(c) Step (b) is iteratively repeated to determine the total length of consecutively aligned segments along the chain.
(d) Using this aligned segment as a reference, neighboring cellulose chains within a radius *r* < 0.5 nm are identified.
(e) For these neighboring chains, the alignment between adjacent $l_{p}$ –long segments is similarly evaluated using *P*_2_(*i*, *i* + 1) and a segment is included as part of the crystallite if it satisfies the criterion from step (b).
(f) Once such an assembly that consists of four or more chains is identified, the total number of monomers contributing to this crystalline domain is recorded.
(g) The entire procedure is repeated for all cellulose chains in the system, excluding those that have already been counted as part of a previously identified crystallite.

Following steps (a–g), the total number of monomers *n*_crystal_ contributing to the crystalline assemblies is counted. The degree of crystallinity is then defined as $d=n_{\mathrm{crystal}}/N_{l}N_{c}$ . Figure 2(b) presents the calculated values of *d* for seven representative samples, together with a couple of snapshots highlighting the chain structure, including a crystalline domain within the sample. In this study, we focus on evaluating κ for all these samples.

We also note in passing that – at first glance – the simulated temperatures may appear unusually high. However, it is important to mention that elevated temperatures (i.e., *T* ≃ 700 K) are routinely used in cellulose processing applications.^41^ Notably, the melting temperature *T*_m_ of cellulose crystals has been reported to lie within the range of 690–753 K.^42^ Furthermore, empirical all–atom models often overestimate the cohesive energy density,^43–45^ which in turn shifts the effective processing temperature range in simulations to higher values compared to experiments.

### 2.2 Glass Transition Temperature

The final equilibrated amorphous configurations are gradually quenched to 280 K using a cooling rate of 1 K/ns. The system is cooled in increments of 50 K down to 400 K, and then in smaller steps of 20 K. During this process, a reduced integration time step of Δ*t* = 0.1 fs is employed. To allow full molecular flexibility, bond constraints are removed throughout the cooling stage, enabling accurate estimation of the enthalpy *H* and mass density ρ_m_. The resulting specific volume *v* = 1/ρ_m_ as a function of temperature *T* is shown in Figure 3 for both cellulose and cellulose acetate. This provides a direct estimate of the glass transition temperature *T*_g_, a key characteristic of amorphous polymers. Based on this analysis, *T*_g_ is estimated to be approximately 625 K for cellulose and 445 K for cellulose acetate. These values are consistent with experimental ranges reported in the literature, which span approximately 476–525 K for cellulose^46,47^ and 370–470 K for cellulose acetate.^48,49^ This discrepancy is expected, as *T*_g_ is highly sensitive to the cooling rate that is typically several orders of magnitude faster in simulations than in experiments. Additionally, the choice of all–atom force field parameters can significantly influence the predicted *T*_g_. For instance, prior studies have shown that *T*_g_ values are often overestimated in simulations of acrylic polymers^45^ and silica.^43^ We note that the precise value of *T*_g_ does not affect κ,^6^ and we present this analysis primarily to validate the reliability of our model.

**Figure 3:**
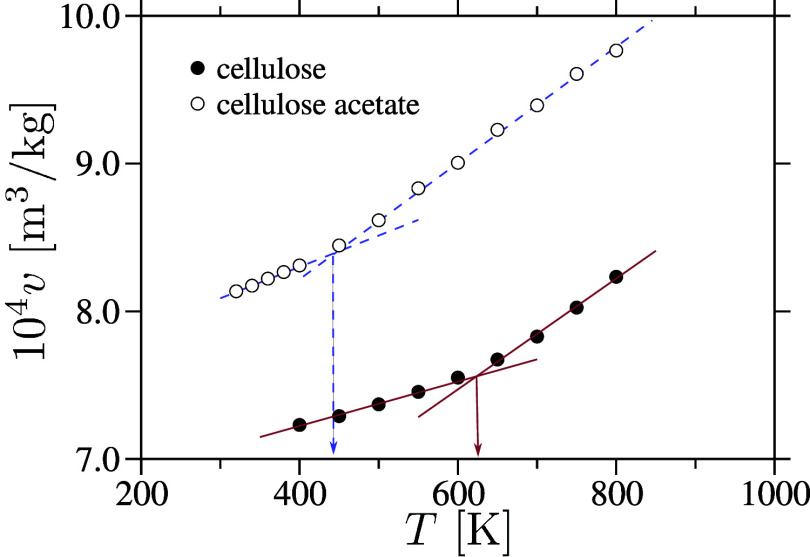
Specific volume, *v*, as a function of temperature, *T*, for amorphous cellulose and cellulose acetate. The glass transition temperatures *T*_g_ is estimated from the crossover points. *T*_g_ are estimated to be approximately 625 K for cellulose and 445 K for cellulose acetate.

All key observables–including the vibrational density of states *g*(ν), enthalpy *H*, heat capacity at constant pressure *c*, components of the elastic modulus tensor *C*_*ij*_, longitudinal $v_{l}$ and transverse *v*_*t*_ sound velocities, and the thermal transport coefficient κ–are calculated within the temperature range of 280 to 400 K.

## 3 Experimental Details of Cellulose Acetate

Samples of cellulose acetate were purchased from Goodfellow as a 50 m thick film. Wide-angle X-ray scattering (WAXS) showed that the samples were fully amorphous. We used displacement thermo-optic phase spectroscopy (D-TOPS) to measure the in-plane thermal conductivity of the films. To prepare the samples for measurement by D-TOPS, we deposited an 80 nm thick Al film by magnetron sputtering. The Al film serves as a transducer in the measurement by absorbing optical power from the pump beam and providing a reflective surface for the probe beam. The electronic thermal conductivity determined by the Weidemann Franz law is 130 Wm^1–^ K^–1^.

The apparatus and procedure is the same as described in ref 50. We employed fiber–coupled superluminescent diodes (SLDs) as light sources, emitting at 780 nm for the pump beam and 670 nm for the probe beam. A motorized actuator adjusted the beam offset by rotating a gimbal–mounted beam splitter. Both beams were focused with a 5× objective lens to a 1/*e* ^2^ intensity radius of 11.2 μs (rms) and the distance between the pump and probe spots was 12.6 μs. The 5× lens was selected to ensure the beam spot size remained at least more than 4 times smaller than the thickness of the cellulose acetate sample. The pump and probe beam powers were 0.65 mW and 0.95 mW, respectively. The calculated steady state temperature rise was 5–7 K for the pump power used for this specific sample. The experimental data of the cellulose acetate sample were analyzed using an isotropic thermoelastic model described in ref 51.

We collected D–TOPS data over the temperature range of 293 ≤ *T* ≤ 373 K by affixing the cellulose acetate sample to a silicon carrier wafer using silver paste and attaching the Si wafer to an Instec temperature–controlled microscope stage. All temperatures reported correspond to the readings from the Instec temperature stage.

Analysis of the D–TOPS data requires knowledge of the heat capacity per unit volume as an input to the model. We determined the specific heat capacity of an approximately 10 mg cellulose acetate sample over a temperature range of 223 to 473 K using a Discover DSC 2500 calorimeter. The obtained specific heat values were then multiplied by the mass density (1300 kg/m^3^) to obtain the volumetric heat capacity.

## 4 General Microscopic Picture of Polymer Thermal Conductivity Revisited

Heat transport in materials is inherently complex, and polymers present additional challenges due to their structural characteristics. In particular, the macroscopic κ of amorphous polymers consisting of linear chains is typically low, reaching a maximum of about 0.4 Wm^–1^ K^–1^ in systems with H–bonding.^24^ Here, energy transport in amorphous polymers occurs through three distinct mechanisms: (i) At the shortest length scales, energy propagates ballistically along the covalently bonded monomeric repeat units of the polymer backbone. (ii) Owing to the random–walk nature of polymer conformations, bends and kinks act as scattering centers, disrupting ballistic motion^12,52,53^ and resulting in diffusive intra–chain transport over distances comparable to a few persistence lengths. (iii) After diffusing along the macromolecular backbone, energy is eventually transferred to a neighboring chain. This inter–chain energy transfer, mediated by weak non–bonded interactions, is the slowest transport mechanism. Nevertheless, it is essential for energy transport over length scales exceeding the polymer’s end–to–end distance, *R*_ee_.^54,55^

The discussions above provide a crucial conceptual point in the calculation of κ in amorphous polymer. In a simple phonon picture, κ in an isotropic system is directly related to the volumetric heat capacity *c*(ν)/*V*, group velocity *v*_g_(ν), and phonon lifetime τ­(ν) = Λ­(ν)/*v*_g_(ν). Here, Λ­(ν) and ν are the phonon mean–free path, and frequency, respectively. Note that this description is not quite rigorous in the case of short relaxation times. More specifically, τ­(ν) is typically very short in amorphous materials, limited to the scale of local atomic vibrations. As a result, heat is primarily transported via local vibrational modes rather than long–wavelength phonons. This is a key reason why κ of amorphous materials is significantly lower – often by several orders of magnitude – than that of their crystalline counterparts. For instance, κ values can exceed 1000 Wm^–1^ K^–1^ in carbon nanotubes^56,57^ and reach around 142 Wm^–1^ K^–1^ in single–crystal silicon at room temperature.^58^ In contrast, amorphous silicon exhibits a much lower κ ≃1.8 Wm^–1^ K^–1^,^59^ while typical non–conducting polymers display values in the range of 0.1–0.4 Wm^–1^ K^–1^.^24,31^

Following the discussion above, the macroscopic κ for the isotropic and nonconducting amorphous materials– within the description of the minimum–thermal–conductivity model– can then be written as^21,31,60^

2 $$\kappa \left(T\right)=\left(\frac{\rho_{N}h^{2}}{6k_{B}T^{2}}\right)\left(v_{l}^{2}+2v_{t}^{2}\right)\int \frac{\nu e^{h\nu /k_{B}T}}{{\left(e^{h\nu /k_{B}T}-1\right)}^{2}}g\left(\nu \right)d\nu$$

ρ_N_(*T*) = *N*/*V*(*T*) is the total atomic number density, *N* the total number of atoms, and *h* the Planck constant. Here, eq 2 relies on two key assumptions: (i) the phonon mean–free path, Λ, is limited to half of the phonon wavelength, yielding τ­(ν) = 1/(2ν). (ii) *v*_*i*_ are the components of the sound–wave velocity, i.e., the longitudinal $v_{l}$ and the transverse *v*_*t*_ sound velocities. While eq 2 typically provides a reasonable estimate of κ, it may be less accurate in systems where the persistence length is significantly larger than the monomer size and/or in the extended chain configurations inducing large local structural asymmetry for energy transfer pathways.

Equation 2 also highlights that calculation of κ at a given *T* requires the vibrational density of states, *g*(ν), the sound–wave velocities, *v*_*i*_, and the number density ρ_N_. For instance, within the typical temperature range used in κ calculations (i.e., 300–400 K, corresponding to frequencies ν ≃ 6.2–8.3 THz), only the low–frequency (soft) modes are thermally excited and contribute significantly to κ. In contrast, high–frequency (stiff) modes in polymers remain largely quantum–mechanically frozen in this temperature window.^30,61^ Nonetheless, vibrational modes up to about 25–30 THz can still contribute marginally, as governed by the Bose–Einstein weighting function in eq 2. In contrast, classical simulations treat all vibrational modes as equally active at *T*, leading to an overestimation of *c* and consequently κ. This discrepancy is one of the primary reasons why κ–values obtained from classical simulations are always overestimated.^31,62^ On the contrary, sound–wave velocities, which reflect the elastic properties of the material, are predominantly dictated by non–bonded interactions in polymers.^31^ It is important to note that we use ν–independent estimates for *v*_*i*_, which is a valid approximation in the low–temperature limit. However, at higher temperatures, this assumption becomes less accurate, and additional considerations may be required. Note that by “low–temperature”, we refer to the range of *T* in which only low–frequency (soft modes) are excited – specifically around 6.2 THz, which corresponds to approximately 300 K.

To incorporate quantum corrections into classically derived κ for polymers, two related approaches have recently been proposed.^31^ One method involves a simple rescaling based on a quantum estimation of the specific heat, *c* ^30^ that gives a lower limit of κ, while the other uses eq 2 directly. In this work, we apply both these approaches to study heat flow in amorphous cellulose and cellulose acetate, whereas for the semi–crystalline samples, we adopt the simpler specific heat rescaling method.

## 5 Results and Discussion

### 5.1 Vibrational Density of States

The vibrational density of states *g*(ν) in our classical simulations is computed using^43,64^

3 $$g\left(\nu \right)=\frac{1}{A}\int_{0}^{\infty }\cos\left(2\pi \nu t\right)\frac{\psi \left(t\right)}{\psi \left(0\right)}dt$$

where the normalization constant *A* ensures that ∫*g*(ν)­dν = 1. The mass–weighted velocity autocorrelation function is defined as ψ­(*t*) = ∑_*i*_ *m*_*i*_⟨*v⃗*_*i*_(*t*)·*v⃗*_*i*_(0)⟩, and represents the superposition of individual normal modes. As a result, its Fourier transform yields *g*(ν), providing insight into the distribution of resonance frequencies across the modes. For the ψ­(*t*) calculations, we have chosen Δ*t* = 0.1 fs and the data is sampled for 10 ps with an output data frequency of 5 × 10^–4^ ps. During this stage, bond constraints were removed and all bonds were allowed to vibrate. Figure 4 displays the calculated *g*(ν) for both samples at *T* = 300 K, where the vibrational peaks that correspond to atomistic vibrations are explicitly labeled.^63^

**Figure 4:**
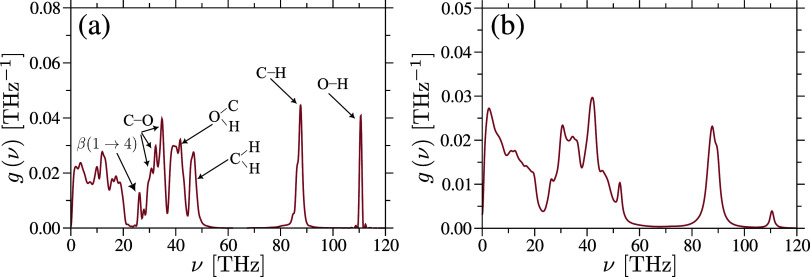
Vibrational density of states *g*(ν) for amorphous cellulose (part a) and cellulose acetate (part b) samples is shown. Characteristic vibrational peaks are labeled in part (a) and are consistent with experimental observations.^63^.

### 5.2 Heat Capacity

We begin by calculating one of the central quantities in our study, *c*(*T*). For this purpose, we will use a recently proposed method in ref 30, which uses the Binder approach of harmonic ref 43 to estimate the difference Δ*c*(*T*) between the classical and the quantum descriptions^30^

$$\frac{\Delta c\left(T\right)}{k_{B}}=\int_{0}^{\infty }\left\{1-{\left(\frac{h\nu }{k_{B}T}\right)}^{2}\frac{e^{h\nu /k_{B}T}}{{\left(e^{h\nu /k_{B}T}-1\right)}^{2}}\right\}g\left(\nu \right)d\nu$$ 4

which is finally used to obtain the quantum–corrected estimate of heat capacity

5 $$c\left(T\right)=c^{\mathrm{cl}}\left(T\right)-\Delta c\left(T\right)$$

Here, *c* ^cl^(*T*) = {*H*(*T* + Δ*T*) – *H*(*T* – Δ*T*)}/2 Δ*T* is the classical estimate of heat capacity and the enthalpy is given by *H*(*T*) = *U*(*T*) + *pV*(*T*). *U*(*T*) is the internal energy and *p* = 1 atm is the external pressure. *c* ^cl^(*T*) is shown by the • data sets in Figure 5. As as expected, comparison of *c* ^cl^(*T*) for cellulose is about a factor of 3 higher than the available experimental data (see line in Figure 5a). Figure 5 also compiles *c*(*T*) for both samples, see eq 5. It is clear that our *c*(*T*) data (represented by the ° data set in Figure 5) is significantly lower than the classical estimates and, in the case of pure amorphous cellulose, aligns reasonably well with the available experimental results.^65^ This agreement further validates our simulation model and protocol, confirming that we accurately capture the key features observed experimentally.

**Figure 5:**
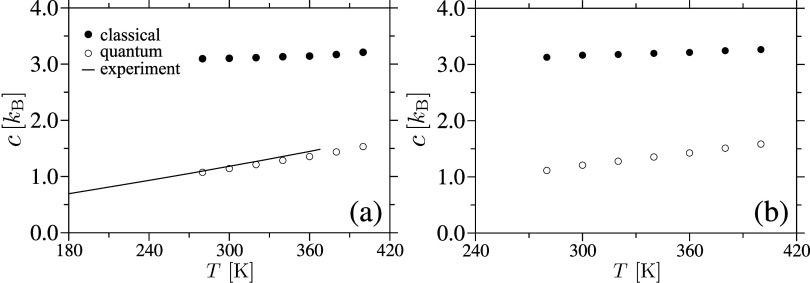
Per atom heat capacity *c*(*T*) as a function of temperature *T* for amorphous cellulose (part a) and cellulose acetate (part b) samples are presented. The data include the classical estimate of heat capacity *c* ^cl^(*T*) (• data sets), and the quantum heat capacity *c* ^qm^(*T*) = *c* ^cl^(*T*) – Δ*c*(*T*) (° data sets). For comparison, available experimental data for amorphous cellulose is also included.^65^.

### 5.3 Thermal Conductivity of Amorphous Cellulose and Cellulose Acetate

#### 5.3.1 Thermal Transport Coefficient Using Classical and Quantum Heat Capacities via the Approach–to–Equilibrium Method

For the computation of κ with varying *T*, we employ the approach–to–equilibrium (ATE) method.^66^ In the ATE approach, the simulation box – of total length *L*_*x*_ along the *x*–direction – is divided into two equal regions, each of length *L*_*x*_/2. One half is maintained at a higher kinetic temperature *T*_Hot_ = *T* + 50 K, while the other is kept at a lower temperature *T*_Cold_ = *T* – 50 K. Initially, both regions are thermalized using canonical simulations for 2 ns with a time step of Δ*t* = 1 fs. Here, *T* is imposed using a Langevin thermostat. After this equilibration, the system is switched to the microcanonical ensemble, allowing the temperature difference Δ*T*(*t*) = *T*_Hot_–*T*_Cold_ to relax naturally over 0.3 ns in our simulation with a smaller time step of Δ*t* = 0.1 fs. Representative data sets for Δ*T*(*t*) for both systems are shown in Figure 6. It can be appreciated that– after an initial fast drop for *t* < 0.05 ns, related to intra–molecular energy transfer– the decay of Δ*T*(*t*) typically follows an exponential form, Δ*T*(*t*) ∝ exp­(−*t*/τ), from which the relaxation–time constant τ is extracted. κ­(*T*) is then computed using the expression provided in ref 66

6 $$\kappa \left(T\right)=\frac{1}{4\pi^{2}}c\left(T\right)\frac{L_{x}\left(T\right)}{A\left(T\right)\tau \left(T\right)}$$

Here, A(T) denotes the cross–sectional area of the samples. Using the classical estimate, *c* ^cl^(*T*), of the specific heat capacity from Figure 5 in eq 6, we compute classical estimate of thermal transport coefficient κ^cl^(*T*), as represented by the • data sets in Figure 7.

**Figure 6:**
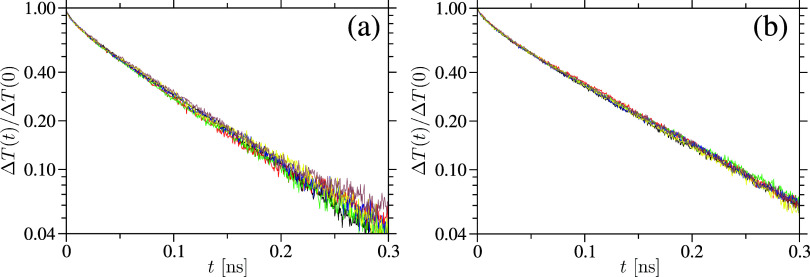
Decay of the temperature difference Δ*T* between the hot and cold domains is shown for amorphous cellulose (part a) and cellulose acetate (part b) samples. An exponential function is fitted in the time window 0.02 < *t* < 0.20 ns to extract the decay time constant, which is then used to calculate the thermal transport coefficient κ. Data is presented for all investigated temperatures. The smaller fluctuations observed in part (b) are attributed to the larger system size of the cellulose acetate sample, which contains approximately 0.3 million more atoms than the cellulose sample.

**Figure 7:**
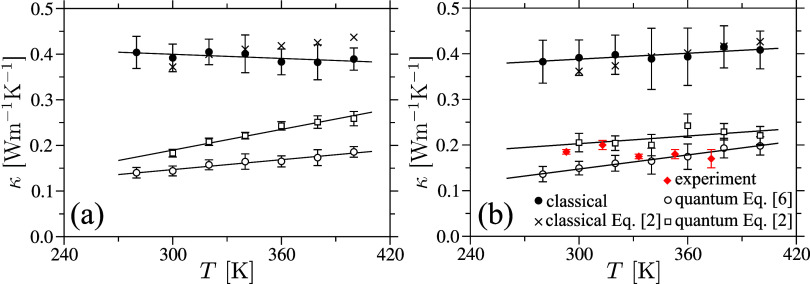
Thermal transport coefficient κ as a function of temperature *T* is shown for amorphous cellulose (part a) and cellulose acetate (part b) samples. The κ values are computed using multiple methods, including both classical and quantum approaches. Error bars for the classical and quantum estimates obtained via eq 6 represent the standard deviations from a set of ten non–equilibrium simulations, each used to determine the decay time constant τ in eq 6. For the quantum estimates based on eq 2, the error bars reflect the standard deviations of κ values calculated using ten independently measured elastic moduli–*C*_11_ and *C*_44_–shown in Figure 8(b),(d). Experimental data for cellulose acetate are also included in part (b). The lines represent linear fits to the data and are provided as visual guides.

While we have performed κ calculations on the larger samples, we have also carried out two test simulations for the pure cellulose systems. In both cases, we used $N_{l}=10$ . The first system consisted of 50 chains in a cubic box with a linear dimension of 4.5 nm. In the second system, we doubled the number of chains to 100, keeping the *x*–and *y*–dimensions at 4.6 nm while extending the *z*–dimension to about 9.0 nm. For the smaller system, we obtained κ ≃0.55 Wm^–1^ K^–1^, whereas for the larger system the value decreased to κ ≃0.32 Wm^–1^ K^–1^. In the smaller system, the typical *R*_ee_ of a chain is comparable to the box size in the smaller system, such that energy transport is dominated by bonded interactions. Note also that the value obtained for the 100–chain system is slightly smaller than in Figure 7(a) at *T* = 300 K, which weakly reflects the dependence of κ on $N_{l}$ .

A previous study identified that the Debye temperatures of H–bonded and vdW commodity polymers typically lie 50–120 K below ambient temperature (*T* = 300 K),^31^ as calculated based on experimental sound wave velocity data.^24^ As a result, the macroscopic heat–transfer time scale, τ, is governed predominantly by the non–bonded (soft) polymer modes, which can be adequately treated classically.^31^ Consequently, one of the primary sources of discrepancy between the actual (i.e., quantum–corrected) thermal conductivity κ­(*T*) and its classical counterpart κ^cl^(*T*) arises from the classical estimate of the heat capacity *c* ^cl^(*T*).^24,30^ In this context, applying quantum corrections of *c*(*T*) in eq 6 yields κ­(*T*) that is smaller than the classical estimates, as demonstrated by the ° data sets in Figure 7.

It has previously been reported in the context of thermal conductivity in commodity polymers that, while applying a quantum correction to the heat capacity – when used in eq 6– generally yields κ values closer to experimental measurements than purely classical estimates,^31^ it can, in some cases, lead to an underestimation of κ relative to certain experimental data.^24^ This discrepancy may arise from contributions due to local structural effects and alternative mechanisms of monomer–level energy transfer– particularly in polymers with persistence lengths significantly larger than the size of individual monomers. Nevertheless, the quantum correction approach using eq 6 captures the leading–order contribution to the difference between classically computed and actual κ values.

In line with the discussion in the preceding paragraph, it has been previously shown that the method based on eq 2 yields results that are in much better agreement with experimental data compared to other approaches.^31^ For instance, in the case of poly­(methyl methacrylate), eq 2 gives κ ≃ 0.21 Wm^–1^ K^–1^,^31^ closely matching the experimental value of κ ≃ 0.20 Wm^–1^ K^–1^.^24^ In contrast, the quantum–corrected heat capacity approach underestimates the thermal conductivity (κ ≃ 0.14 Wm^–1^ K^–1^), while the classical method significantly overestimates it (κ ≃ 0.31 Wm^–1^ K^–1^). Therefore, we next apply eq 2 to compute the thermal conductivity κ of amorphous cellulose and cellulose acetate.

#### 5.3.2 Thermal Transport Coefficient Using the Exact Vibrational Density of States

Equation 2– previously applied to compute κ for various synthetic polymers^31^– requires four key input parameters: (i) the total number density of atoms, ρ_N_; (ii)­the longitudinal sound velocity, $v_{l}$ ; (iii) the transverse sound velocity, *v*_*t*_; and (iv) the vibrational density of states, *g*(ν). Provided these quantities are estimated with reasonable accuracy, κ­(*T*) can be computed. The longitudinal and transverse sound velocities are given by $v_{l}=\sqrt{C_{11}/\rho_{m}}$ and $v_{t}=\sqrt{C_{44}/\rho_{m}}$ , respectively, where ρ_m_ denotes the mass density.

To compute *C*_11_ and *C*_44_, we employ a recently developed method^67^ that enables accurate determination of the elastic modulus tensor components, *C*_*ij*_, at finite temperatures. This method mitigates thermal noise using a noise–cancellation technique originally introduced for calculating piezoelectric coefficients in crystalline silica.^68^ Specifically, the shear modulus *C*_44_ is computed by applying a strain ε to a cubic simulation box of length *L* in a volume–conserving fashion, using the following transformation

7 $$L_{x}=L\left(1+\varepsilon \right),L_{y}=\frac{L}{1+\varepsilon },L_{z}=L$$

Here, *L*_*i*_ denotes the box lengths along the *x*, *y*, and *z* directions. For small strains (we use ε = 10^–3^), and assuming cubic symmetry, the shear modulus is given by

8 $$C_{44}=\frac{\sigma_{\mathrm{xx}}\left(+\varepsilon \right)-\sigma_{\mathrm{xx}}\left(0\right)}{2\varepsilon }\;\mathrm{or}\;C_{44}=-\frac{\sigma_{\mathrm{yy}}\left(-\varepsilon \right)-\sigma_{\mathrm{yy}}\left(0\right)}{2\varepsilon }$$

where σ_*ij*_ are the components of the stress tensor.

To obtain *C*_11_ and *C*_12_, we apply uniaxial strains along the *x*–direction in two separate simulations: *L*_*x*_ = *L*(1 ± ε), while keeping all other strain components zero. Taking the difference between the stress responses of these oppositely strained configurations yield

9 $$C_{11}=\frac{\sigma_{\mathrm{xx}}\left(+\varepsilon \right)-\sigma_{\mathrm{xx}}\left(-\varepsilon \right)}{2\varepsilon }\;\&\;C_{12}=\frac{\sigma_{\mathrm{yy}}\left(+\varepsilon \right)-\sigma_{\mathrm{yy}}\left(-\varepsilon \right)}{2\varepsilon }$$

Figure 8(a) shows the time convergence of the elastic constants *C*_*ij*_ for one amorphous cellulose sample at *T* = 300 K. The average values of *C*_11_, *C*_12_, and *C*_44_ for amorphous cellulose and cellulose acetate samples are computed within the time window 50 ≤ *t* ≤ 150 ps, as illustrated in panels (b–d) of Figure 8. As expected, *C*_*ij*_ decrease with increasing *T*, indicating a softening of the samples. We also note that *C*_11_ can be expressed in terms of *C*_44_ and the Poisson’s ratio ν_p_ as *C*_11_ = 2*C*_44_(1 – ν_p_)/(1 – 2ν_p_). Figure 8(e) presents a plot of *C*_11_ versus *C*_44_, along with two reference lines corresponding to ν_p_ = 0.35 and 0.40– typical values for vdW and H–bonded polymers.^28^ Notably, the simulation data in Figure 8(e) fall within the bounds set by these reference lines, further validating the accuracy of our estimated elastic constants *C*_*ij*_.

**Figure 8:**
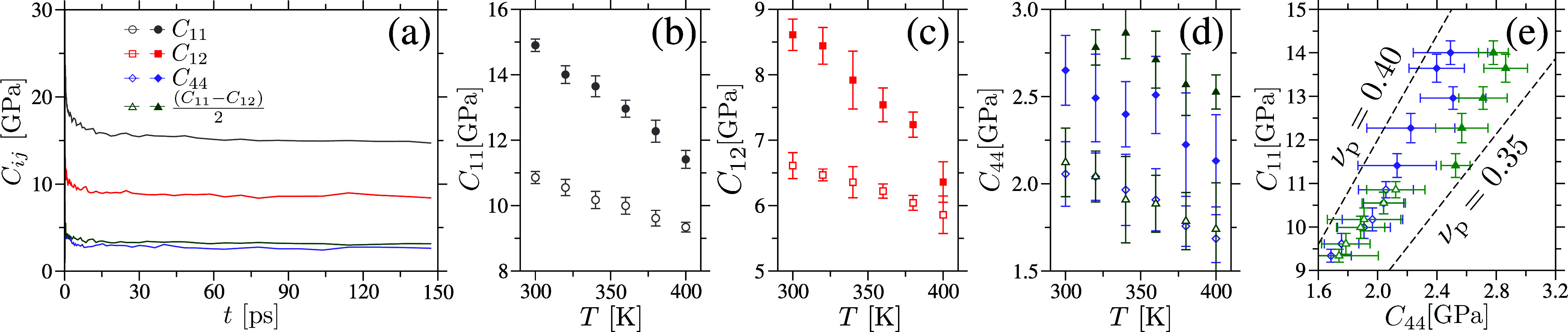
Panel (a) displays the time convergence of the elastic constants *C*_*ij*_ for an amorphous cellulose sample at a temperature *T* = 300 K, calculated using eqs 8 and 9. The average values of *C*_ij_ as a function of *T*, shown in panels (b–d), are computed by averaging the data over the time interval 50 ≤ *t* ≤ 100 ps. Errorbars indicate the standard deviation, determined from five separate 2 ps time windows within this interval. The solid symbols represent amorphous cellulose, while the open (empty) symbols correspond to cellulose acetate samples. Panel (e) presents the relationship *C*_11_ = 2*C*_44_ (1 – ν_p_)/(1 – 2ν_p_), with two reference lines corresponding to the Poisson’s ratios ν_p_ = 0.35 and 0.40, highlighted within the panel. These lines, with slopes 2­(1 – ν_p_)/(1 – 2ν_p_), serve as benchmarks for comparison with typical values observed in H–bonded and vdW–based polymers.^28^.

The *C*_11_ and *C*_44_ values from Figure 8(b),(d), along with *g*(ν) from Figure 4 are used in eq 2 to calculate κ­(*T*). The resulting values are represented by the □ data set in Figure 7. While the overall trend– namely, the increase in κ with *T*– is captured, the absolute κ values obtained from eq 2 are approximately 20–25% higher than those derived from the calculations using eq 6. A possible source of deviation lies in the limitations of the simple scaling approach used in eq 6, which accounts only for corrections to the specific heat. However, additional factors, such as local (monomer–level) variations in energy transfer mechanisms, may also play a non–negligible role.

To the best of our knowledge, temperature–dependent experimental data for dense amorphous cellulose and cellulose acetate are currently unavailable. Therefore, we have also performed experimental measurements on cellulose acetate, which is known to form amorphous samples. The corresponding results are shown as the red ⧫ in Figure 7(b), where a reasonable agreement between our simulation and experimental data is clearly evident. We also note in passing that obtaining a purely amorphous cellulose sample is rather nontrivial, as such a system have a strong tendency to crystallize rapidly. Therefore, we do not have experimental κ for the pure amorphous cellulose sample.

### 5.4 Thermal Conductivity of Semi–Crystalline Cellulose

A straightforward strategy for tuning κ of a polymeric material is to modify *d* in polymers that tend to form crystalline domains. Typical examples include, but are not limited to, polyethylene fibers,^52^ cellulose fibers,^12^ poly­(vinyl alcohol),^69^ thermoelectic polymers,^70^ organic semi–conductors,^23^ polypeptide sequences,^71^ and/or semi–crystalline epoxies.^40,72^ In these materials, crystalline order– and consequently larger κ– can arise from the regular arrangement of bonded monomers,^12,52,53^ π–π stacking,^23,70^ H–bonded stacking,^69^ or secondary structure of polypeptides.^71^ Regenerated cellulose,^73,74^ in particular, offers a natural platform for producing samples with varying *d*, where d–glucose stacking promotes crystalline ordering. Accordingly, in the following, we investigate the behavior of κ in semi–crystalline samples, as discussed in Figure 2.

Figure 9 shows κ for semi–crystalline samples as a function of *d*. As expected, it can be appreciated that κ increases by about 35%. At first glance, this may seem like a modest increase. However, a semi–crystalline sample consists of crystallites embedded in an amorphous matrix. While energy transfer within the crystalline domains occurs more efficiently, the surrounding amorphous regions act as resistance to the heat flow, thereby limiting the increase in macroscopic κ. A detailed discussion on the semi–crystalline and crystalline cellulose samples will be presented elsewhere.

**Figure 9:**
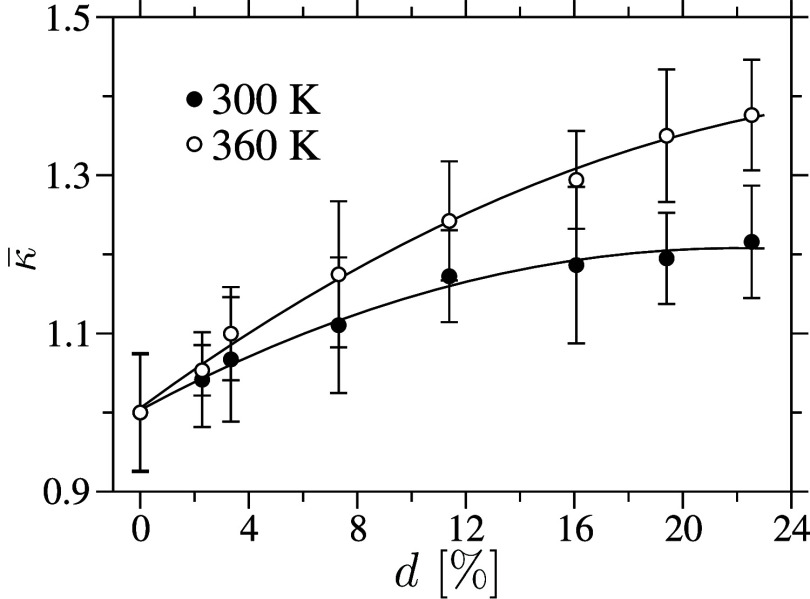
Normalized thermal transport coefficient κ is shown as a function of the degree of crystallinity *d* for semi–crystalline samples. Data is presented for two different temperatures *T*, with κ values normalized relative to those of the corresponding amorphous samples, as shown in Figure 7. Error bars indicate the standard deviations of κ, calculated from a set of ten nonequilibrium simulations used to determine the time constant τ in eq 6. The lines are included as visual guides to aid the eyes.

### 5.5 Some General Discussions on Cellulose Thermal Conductivity and Possible Tunability by Macromolecular Engineering

Our reported κ values for a few cellulose samples are comparable to that of the common synthetic polymers.^24,31,75^ For instance, amorphous cellulose has a mass density of approximately ρ_m_ ≃ 1390 kg/m^3^ and quantum–corrected κ within the range of 0.14–0.18 Wm^–1^ K^–1^ at *T* = 300 K (see Figure 7). In comparison, poly­(acrylic acid) (PAA) – a polymer whose properties are largely governed by H-bonds – exhibits nearly twice as large κ ≃0.37 Wm^–1^ K^–1^,^24^ while maintaining a similar density ρ_m_ ≃1250–1410 kg/m^3^.^28^ Another example, poly­(methyl methacrylate) (PMMA), dominated by vdW interactions, has a lower density ρ_m_ ≃ 1200 kg/m^3^ but a comparable κ ≃ 0.12–0.20 Wm^–1^ K^–1^ ^24,31,75^ to amorphous cellulose. Furthermore, *C*_11_ and the shear modulus *C*_44_ of PAA are approximately 20–25% higher than those of amorphous cellulose, while those of PMMA are of similar magnitude. Notably, both PAA and PMMA chains exhibit significantly lower bending stiffness – quantified by persistence length $l_{p}≃0.65\text{−}0.75\;\mathrm{nm}$ ^28,29,55^ –compared to cellulose $l_{p}=3.4\;\mathrm{nm}$ . This reduced stiffness hinders local interchain stacking and limits the extent of local chain stretching, both of which tend to increase κ.

From the above discussion, it is evident that heat transport in polymers – particularly in cellulose – is governed by a complex interplay between different factors, including monomer structure, monomer–monomer interactions, chain conformation and stiffness, as well as overall morphology. In cellulose, the d–glucose monomer introduces additional complexity due to a delicate balance of correlations both within the monomer and between bonded and non–bonded neighboring units. A clear illustration of the importance of monomer structure emerges when polymer chains are stretched into fibers; a regime where bonded interactions dominate κ. For example, the reported κ values for polyethylene fibers, composed of a relatively simple monomer structure, range between 20 Wm^–1^ K^–1^ ^76^ and 100 Wm^–1^ K^–1^.^52^ In contrast, cellulose fibers have reported a maximum of about 14.5 Wm^–1^ K^–1^.^12^ This difference in κ between polyethylene and cellulose fibers is largely attributed to the d–glucose units, which act as intrinsic phonon–scattering centers. Moreover, heat dissipation occurs not only along the backbone but also through vdW interactions between stacked cellulose chains and via H–bonds between the hydroxyl side groups, further limiting the efficiency of thermal transport.

While the κ values reported in this study are reasonable within the context of standard commodity plastics, it is important to explore strategies for tuning κ in cellulose derivative– particularly with the goal of enhancing it. Two promising approaches include: (i) increasing mechanical stiffness, characterized by the elastic constants *C*_11_ and *C*_44_; and (ii) engineering the macromolecular structure of cellulose to enhance the population of low–frequency vibrational modes, which contribute significantly to thermal transport at ambient temperatures.

In terms of increasing stiffness, insights can be drawn from H–bond–dominated polymers such as poly­(acrylic acid) (PAA). PAA exhibits a higher cohesive energy density than vdW–dominated polymers like poly­(methyl methacrylate) (PMMA), due to its strong intermolecular hydrogen bonding. In cellulose, however, the dominant intermolecular interactions are vdW forces – primarily between d–glucose units.^2,3,7^ This interaction is one of the key reasons cellulose is insoluble in most common solvents.^9,73,74^ Nevertheless, cellulose can be chemically modified to tailor its properties for specific applications. One such modification is cellulose acetate (see Figure 1b), which is also examined in this work. Another notable derivative is carboxymethyl cellulose (CMC), where the side group is −CH_3_COONa in the charged form and −CH_3_COOH in the neutral form. The introduction of such relatively bulky side groups disrupts the stacking of d–glucose units, thereby preventing crystallization and maintaining an amorphous structure–an attribute often desired in the design of flexible plastics. In this context, our results show that although cellulose and cellulose acetate exhibit very similar thermal conductivity values–with cellulose acetate having a slightly lower κ– it also has an approximately 10% lower mass density, while maintaining a similarly populated vibrational density of states (see Figure 10).

**Figure 10:**
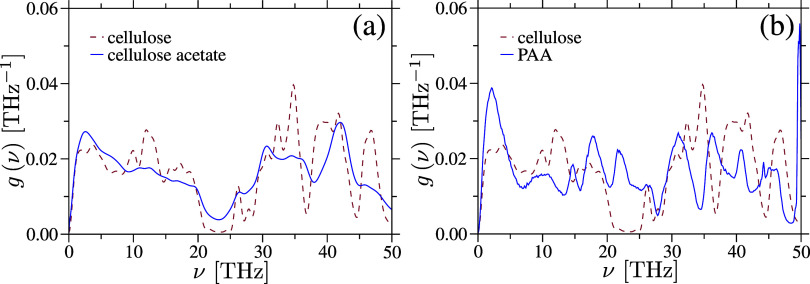
Comparison of the vibrational density of states of cellulose with cellulose acetate (part a) and with poly­(acrylic acid) (PAA) (part b). For the clarity of presentation, only the low frequency range of ν = 0–50 THz is highlighted. Data for PAA are taken from ref 31.

A more promising modification could involve grafting short PAA oligomers as side chains along the cellulose backbone. Such PAA–modified cellulose is expected not only to enhance the elastic moduli but also to increase the population of low-frequency vibrational modes. The latter is highlighted in Figure 10, where a clear enhancement of *g*(ν) can be observed in PAA compared to cellulose. A detailed investigation of different side–chain modifications will be presented elsewhere.

## 6 Conclusions and Outlook

Using large–scale molecular dynamics simulations of an all–atom GLYCAM06 model,^32,33^ we have investigated the thermal conductivity κ behavior of solvent–free, dense amorphous cellulose, cellulose acetate and selected semi–crystalline cellulose samples. Our simulations involved system sizes 0.63–0.93 million particles and a total accumulated simulation time of over 11 μs. The thermal transport properties of cellulose–based materials– such as aerogels with varying porosity,^14–17^ papers,^13^ networks,^25^ nanocrystal assemblies with liquid crystalline order,^26^ and, more recently, fibers^12^– have been experimentally studied. Our work focuses on a comparatively underexplored systems. This investigation is particularly relevant given the ongoing efforts to develop environmentally friendly polymeric materials for their potential suitability for a broad range of applications. The typical applications are likely to include, but are not limited to, packaging of eco–friendly devices,^77^ bio–compatible food wraps, and/or common kitchen utensils.

To compute κ, we employed two modeling approaches^30,31^ that account for the contributions of stiff polymer modes– via its vibrational density of states *g*(ν)– which remain quantum mechanically frozen at ambient temperature *T* conditions and therefore do not contribute to macroscopic thermal transport. These methods allow our simulations to more reasonably replicate experimental conditions. We note in passing that any meaningful comparison between simulation and experimental observations of polymer thermal conductivity– especially across a range of thermodynamic state points– requires such an approach. In contrast, classical calculations of κ often suffer from uncontrolled artifacts that hinder reliable interpretation. In particular, our findings show that as the system transitions from amorphous to semi–crystalline states– with varying degrees, *d*, of crystallinity and *T*– κ spans the range of 0.12–0.24 Wm^–1^ K^–1^. In particular, the simulated amorphous cellulose acetate samples show κ ≃ 0.12–0.22 Wm^–1^ K^–1^, while the complementary experimental κ ≃0.15–0.21 Wm^–1^ K^–1^. These values fall well within the range reported for synthetic polymers.^24,75^ Thus, cellulose can be regarded as a viable alternative.

It will certainly require additional experimental and simulation studies to validate our results. However, given a consistent picture presented in this study, our work is likely to serve as a guiding path for the future studies within in the context of thermal properties of amorphous and semi–crystalline cellulose materials.

## Data Availability

The scripts and the data associated with this research is available upon reasonable request from the corresponding author.
